# Association Between Postoperative Delirium and Loss of Melatonin Rhythm After Hip Arthroplasty: A Pilot Study

**DOI:** 10.7759/cureus.86728

**Published:** 2025-06-25

**Authors:** Julio C Pérez-Alavéz, Jesús T Castañeda-López, Andrea G Nieto-Nino, Adelina Rojas-Granados, Manuel Angeles-Castellanos

**Affiliations:** 1 Orthopedic Service, Grupo Angeles Hospital, Mexico City, MEX; 2 Orthopedic Service, Xoco General Hospital, Mexico City, MEX; 3 Anatomy, Faculty of Medicine, Universidad Nacional Autonoma de Mexico, Mexico City, MEX

**Keywords:** circadian rhythm, delirium, hip fracture, melatonin, old age

## Abstract

Introduction: The development of in-hospital postoperative delirium is one of the most common adverse complications in older patients. It can complicate the clinical course and prognosis, leading to patient deterioration. Its incidence is high, affecting up to 55% of patients undergoing procedures such as hip arthroplasty.

Material and methods: Thirty-four hospitalized patients aged 60 years and older with a diagnosis of hip fracture, but without delirium at admission, were included in the study. Daytime and nighttime saliva samples were collected one day before and one day after surgery. Pre- and postoperative melatonin levels were analyzed. Patients were then divided into two groups for comparison: those who developed delirium and those who did not.

Results: Postoperative delirium was observed in 35.29% of patients. In the control group, salivary melatonin levels showed low concentrations during the day and high levels at night, indicating a preserved daily rhythm in melatonin secretion (F(1,33) = 82.639; p < 0.001). This pattern was consistent before and after surgery. In contrast, the delirium group also showed a diurnal rhythm with low morning and high evening melatonin levels before and after surgery, but without a statistically significant difference (F(1,22) = 0.225; p = N/A).

Conclusions: This study concludes that assessing melatonin levels during both light and dark phases is important to determine whether the circadian rhythm of melatonin secretion has been disrupted. This information may help guide the use of melatonin to prevent or anticipate the development of postoperative delirium.

## Introduction

The development of in-hospital postoperative delirium is one of the most common adverse complications in older patients. It can complicate the clinical course and prognosis, potentially leading to deterioration and even death [1].

Delirium is a neuropsychiatric syndrome characterized by severe impairment of mental abilities, particularly inattention and cognitive dysfunction. It may also involve decreased alertness, hallucinations, delusions, mood swings, and severe behavioral disturbances. Delirium has a prevalence of 11%-42% among hospitalized elderly patients annually [2-4], and an incidence of 35%-55% has been reported in patients undergoing hip arthroplasty [5].

Although delirium is not considered a direct complication of orthopedic surgery, its strong association with such procedures has gained increasing attention in recent years [6-8]. Studies have shown that elderly patients undergoing orthopedic surgery have a risk of developing delirium more than three times higher than that of other patients [9].

Some studies have also examined the association between the type of anesthesia and perioperative medications with an increased likelihood of postoperative delirium [10].

Patients with delirium often experience a reversal of the sleep-wake cycle. They tend to be drowsy and sleep during the day, while nighttime sleep is disrupted and fragmented [11-13]. Melatonin is typically released during the dark phase of the circadian cycle, relying on effective communication between the suprachiasmatic nuclei and the pineal gland [14]. Interestingly, some studies have shown that melatonin levels can be altered after surgery. For example, patients who developed delirium following major abdominal surgery exhibited reduced plasma melatonin levels and sleep disturbances, such as delayed sleep phase disorder or inversion of the sleep-wake cycle [11,15,16].

We have previously shown that in hospitalized patients who develop delirium, there is often either a loss of circadian rhythm in melatonin secretion or significantly decreased average melatonin levels starting three days before the onset of delirium, suggesting a possible link between delirium and disruption of melatonin rhythm [14]. This supports the potential preventive use of exogenous melatonin in surgical settings [16]. Therefore, the primary objective of this study was to evaluate whether the loss of circadian rhythm in melatonin secretion is associated with the development of postoperative delirium in elderly patients undergoing hip arthroplasty.

## Materials and methods

This study was approved by the Ethics Committee of Xoco General Hospital (No. 207-010-21-19) and conducted in the Traumatology and Orthopedics Department of Xoco General Hospital, Mexico City, from December 2019 to September 2024, although it was suspended during the COVID-19 pandemic (2020-2022) due to the risks associated with salivary sampling.

The sample size was calculated using G*Power (Ver. 3.1 Heinrich-Heine-Universität Düsseldorf, Düsseldorf, Germany), applying a fixed-effects, omnibus, one-way ANOVA. A total of 44 subjects (22 per group) were required to detect a large effect size. However, due to the study conditions, only 34 patients were enrolled (12 in the delirium group and 22 in the control group).

Patients aged 60 years or older, hospitalized without a diagnosis of delirium upon admission, who signed an informed consent form, and who were diagnosed with a hip fracture or scheduled for surgical treatment were included in the study. Patients unable to speak or understand, on mechanical ventilation or receiving sedative-relaxant medications, with concomitant use of melatonin, and those with structural brain pathology or any condition associated with delirium development, such as sleep disorders, severe pre- or postoperative pain, or psychiatric conditions like anxiety or depression, were excluded. Patients who chose to withdraw from the study, refused saliva sampling for any reason, or for whom saliva collection became unfeasible were eliminated from the study.

All participants were assessed for delirium risk one day before and one day after surgery, based on DSM-IV-TR diagnostic criteria.

Groups

**Figure 1:**
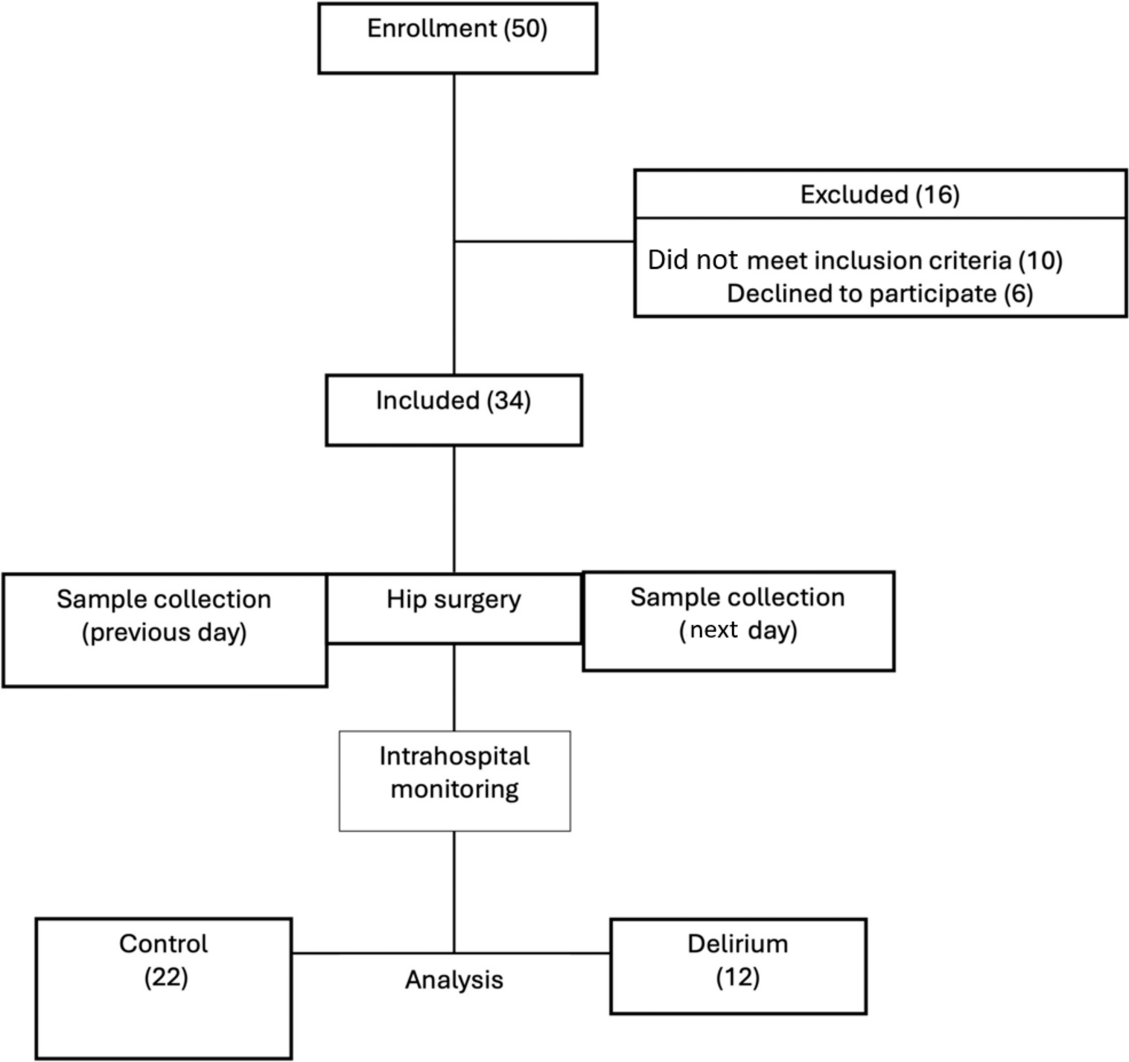
CONSORT diagram of the study

All patients included in the study showed no clinical signs or risk factors for developing delirium upon admission or prior to surgery. Therefore, group classification was done retrospectively, and patients were subsequently divided into the delirium group (D) and the control group (C) (Figure 1).

Lighting conditions

The illumination in the hospitalization area was 151 ± 15.8 lux during the day (7:00 a.m. to 7:00 p.m.), provided by light provided by white fluorescent ceiling lamps, and 40.28 ± 10 lux at night (7:00 p.m. to 7:00 a.m.).

Melatonin determination

Saliva samples were collected from all patients one day before and one day after surgery, at two time points, morning (7:00 a.m.) and evening (9:00 p.m.). Saliva samples were obtained from the oral cavity using a disposable pasteurized pipette and stored under refrigeration until analysis. Salivary melatonin concentrations were measured using an ELISA kit for the direct quantitative determination of melatonin in human saliva (IBL International, Cat. No. RE54041).

Statistical analysis

Age differences between groups were analyzed using an independent samples t-test. Melatonin concentrations, treated as repeated measures, were compared using a two-way ANOVA followed by a Tukey post hoc test. A p-value of < 0.05 was considered statistically significant. Statistical analyses were performed using Statistica version 4.5 (TIBCO Software Inc., Palo Alto, CA, USA), and graphs were generated using SigmaPlot version 14.0 (Grafiti LLC, Palo Alto, CA, USA).

## Results

During the study, a total of 50 patients were enrolled, of whom 16 were excluded. Ultimately, 34 patients were included and divided into two groups: the control group (C; n = 22) and the delirium group** **(D; n = 12) (Figure 1).

**Figure 2:**
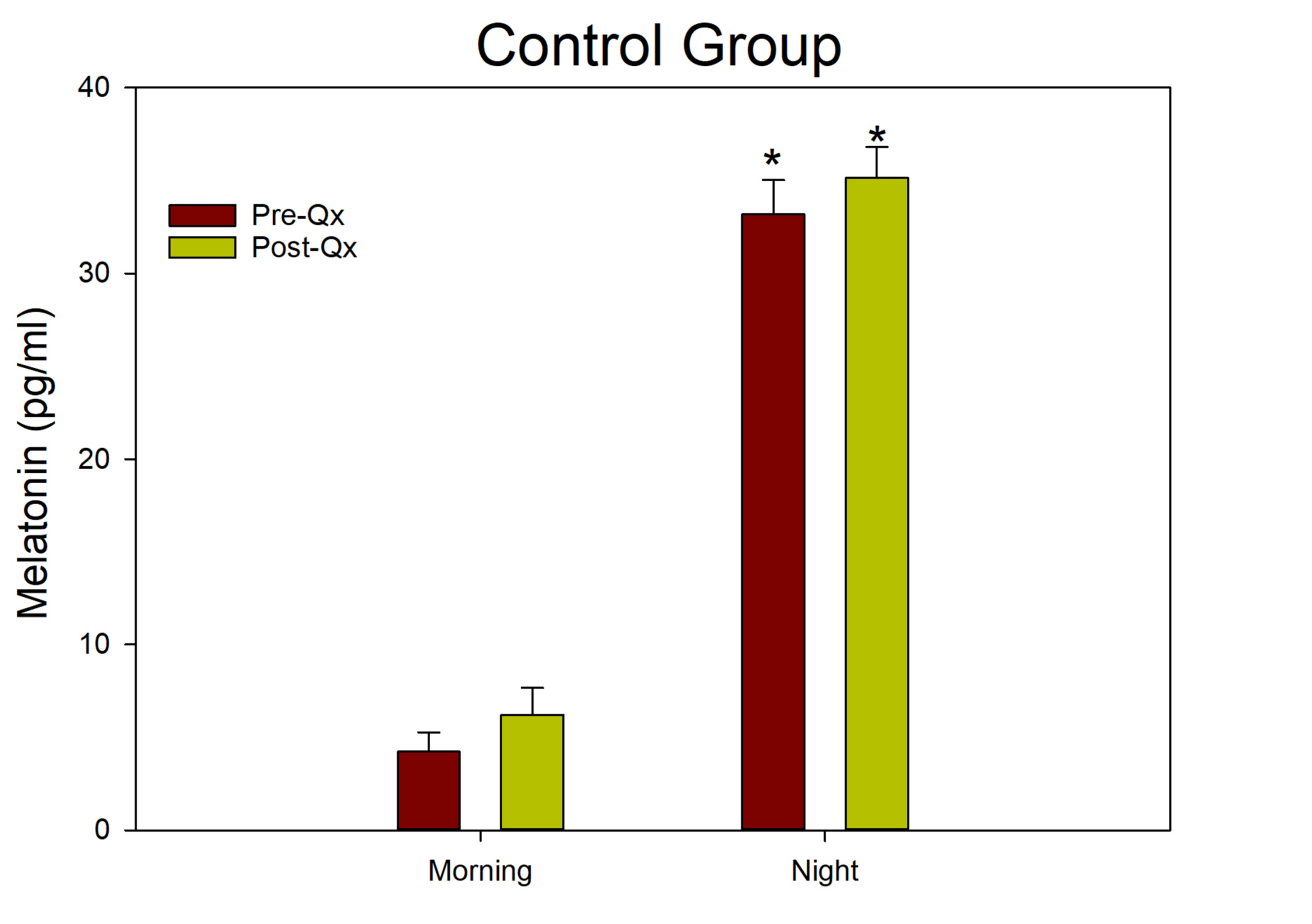
Salivary melatonin levels before and after surgery in the control group. The ANOVA indicated significant differences between morning and evening melatonin concentrations (F(1,33) = 82.639; p < 0.001)

Among the study population, 35.29% developed postoperative delirium, while 64.71% remained stable. The mean age was 83 ± 1.98 years in the C group and 80 ± 1.73 years in the D group, with no statistically significant difference (p = N.S.). All patients had a diagnosis of hip fracture requiring elective surgical treatment; this factor did not influence group differences in outcomes. In the C group, salivary melatonin levels were low during the day and high at night, demonstrating a circadian rhythm in melatonin secretion (Figure 2).

**Figure 3:**
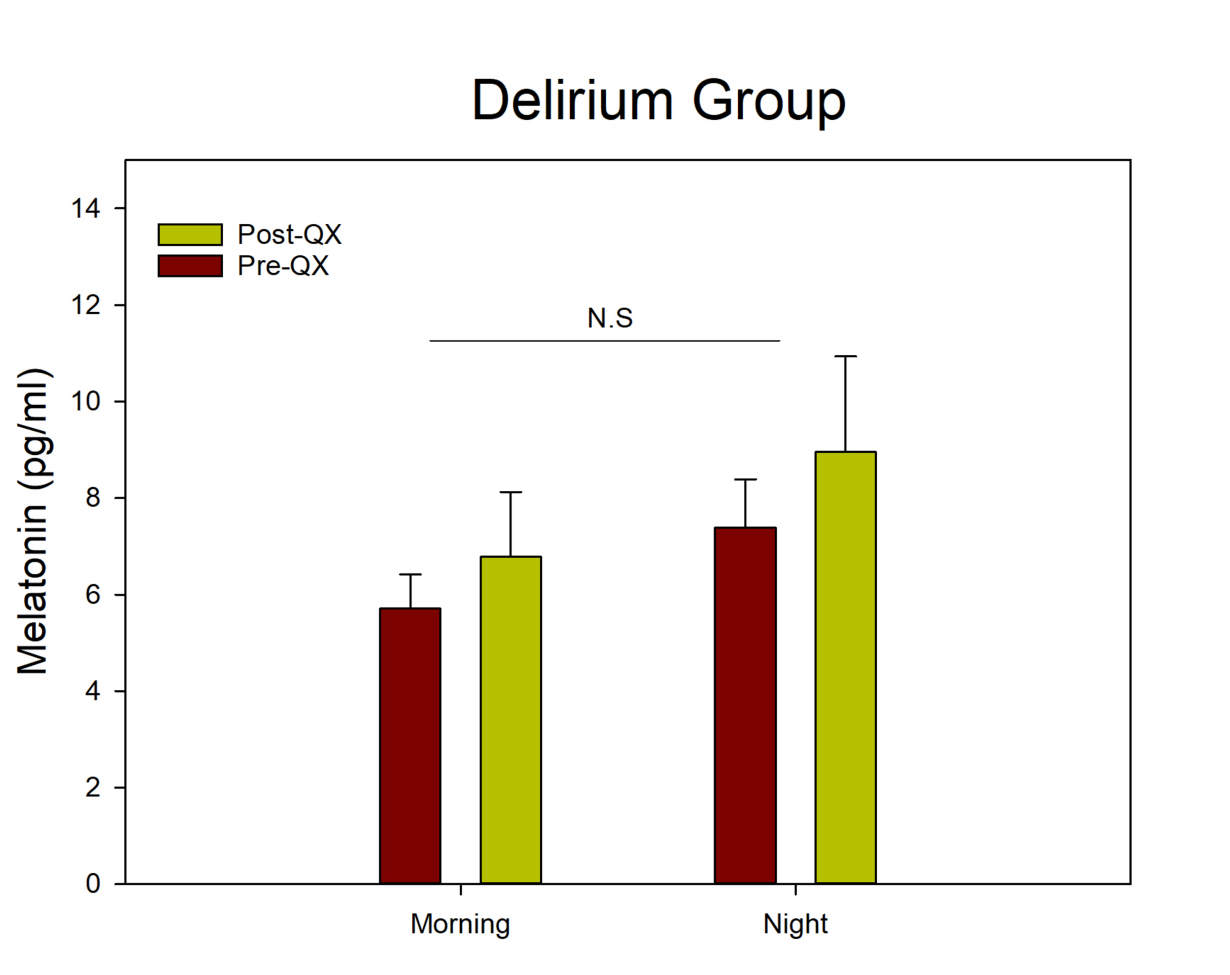
Salivary melatonin levels before and after surgery in the delirium group. The ANOVA showed no significant differences between morning and evening melatonin concentrations (F(1,22) = 0.225; p = N.S.)

The ANOVA indicated significant differences between the light and dark phases (F(1,33) = 82.639; p < 0.001), both before and after surgery, confirming a preserved daily rhythm in melatonin secretion. In contrast, group D showed similar melatonin levels in the morning and evening, with consistently low concentrations before and after surgery, indicating a lack of circadian variation. The ANOVA revealed no significant differences between daytime and nighttime values ​​(F(1,22) = 0.225; p = N/A) (Fig. 3).

**Figure 4:**
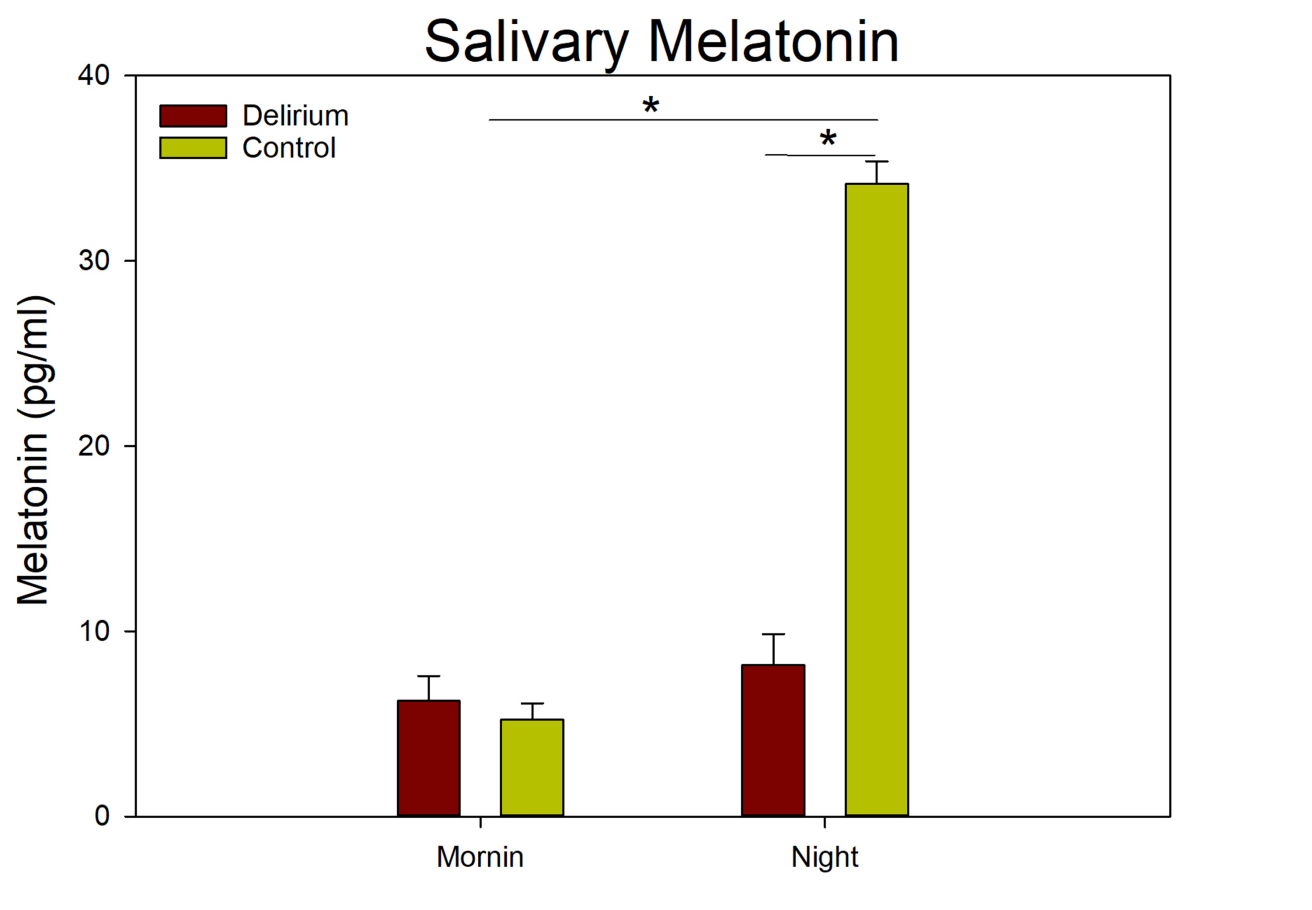
Salivary melatonin levels before and after surgery in the control (green) and delirium (brown) groups. The ANOVA indicated significant differences between day and night melatonin concentrations and a statistically significant difference between groups and time (F(4,66) = 17.447; p < 0.001)

In patients who developed delirium, melatonin levels were low and showed no diurnal rhythm even before the onset of delirium. In contrast, patients who did not develop delirium maintained rhythmic melatonin patterns before and after surgery. A two-way ANOVA assessing the effects of group and time revealed significant differences between groups (F(1,132) = 94.52; p < 0.001), a significant effect of time (F(1,132) = 144.54; p < 0.001), and a significant interaction between group and time (F(1,132) = 110.73; p < 0.001) (Figure 4).

## Discussion

The development of delirium, whether postoperative or in critically ill patients, is associated with increased morbidity and prolonged hospital stays. More recently, it has also been identified as an independent predictor of mortality. Reported incidence rates of delirium in hospitalized patients range from 11% to 42% [1,16]. The results of this study suggest a possible relationship between delirium and the loss of melatonin rhythm, findings that are consistent with our previous report in hospitalized patients [11]. We observed that individuals who developed postoperative delirium already exhibited a loss of diurnal melatonin rhythm before surgery. This aligns with earlier findings showing that patients who developed in-hospital delirium had disrupted daily melatonin rhythms at least three days prior to symptom onset [17]. In this study, the incidence of postoperative delirium was 35.29%, consistent with previously reported rates of 35%-55% in high-risk procedures such as hip fracture repair [5]. While several studies have explored factors contributing to postoperative delirium, including the type of surgery, anesthesia, and analgesia, some have reported much lower incidences of 2.5% to 3.1% [6,10]. Although it was suggested that the lower incidence of postoperative delirium was likely due to the use of regional anesthesia, postoperative opioids, and early ambulation, the characteristics of the study population should also be considered. Importantly, the participants were not limited to older adults, who are the most at risk of developing delirium [3]. Melatonin supplementation has been proposed as a preventive strategy for delirium. Some reports suggest it can reduce the incidence of in-hospital delirium in older adults by more than 50% [18-20]. However, studies focusing specifically on the postoperative period are limited and show inconsistent results, ranging from no effect to a 70% reduction in incidence [21]. A key limitation of previous studies is the lack of melatonin level monitoring at critical time points, namely, during both the dark and light phases. Most studies administered melatonin without first determining whether patients had a disrupted melatonin rhythm or needed supplementation. Our study addresses this gap by directly measuring salivary melatonin levels, and our findings suggest a possible link between delirium and the loss of melatonin rhythmicity. Given that delirium is a clinical syndrome requiring early recognition for better outcomes [20], we believe it is crucial to first assess whether the circadian rhythm of melatonin secretion is disrupted. Identifying this early could help anticipate the development of postoperative delirium and guide preventive strategies such as targeted melatonin administration.

The limitations of our study include the small sample size and challenges encountered at the beginning of the study due to the COVID-19 pandemic, which restricted and prolonged the patient recruitment period.

## Conclusions

This study suggests that assessing melatonin levels during both the dark and light phases is important for identifying a loss of circadian rhythm in melatonin secretion. This may help determine whether melatonin supplementation could be used to prevent, or at least anticipate, the development of postoperative delirium.
